# Uniportal thoracoscopic lobectomy for intralobar pulmonary sequestration

**DOI:** 10.1186/s13019-016-0425-z

**Published:** 2016-02-11

**Authors:** Alan D. L. Sihoe, Qigang Luo, Guangqiang Shao, Yue Li, Jinglong Li, Dazhi Pang

**Affiliations:** Division of Thoracic Surgery, Department of Surgery, The University of Hong Kong Shenzhen Hospital, Shenzhen, China

**Keywords:** Intralobar, Lobectomy, Pulmonary sequestration, Single port, Thoracoscopic surgery, Uniportal, Video Assisted Thoracic Surgery (VATS)

## Abstract

**Background:**

Pulmonary sequestration is an uncommon congenital condition for which surgical resection is usually indicated – either via open thoracotomy or conventional multi-port Video-Assisted Thoracoscopic Surgery (VATS). Of the two types of sequestration, intralobar sequestration is technically more challenging to resect.

**Case Presentation:**

We report the management of a 34 year old male patient with a long history of respiratory symptoms, and an extensively diseased right lower lobe. A diagnosis of sequestration was confirmed by CT scanning, showing three separate anomalous feeding vessels arising from the abdominal aorta. A right lower lobectomy using a Uniportal VATS approach was performed, and the patient discharged home on the fourth post-operative day.

**Conclusion:**

This is the first report to our knowledge demonstrating the safety and feasibility of the Uniportal approach for the resection of a relatively challenging intralobar sequestration.

## Background

Pulmonary sequestration is an uncommon congenital condition in which a part of the lung becomes separated from the caudal foregut during embryonic development, resulting in that part of the lung having no bronchial communication with the normal tracheobronchial tree [1–3]. Sequestration is characterized by the abnormal lung receiving its arterial blood supply from one or more abnormal vessels that usually arise from the descending thoracic aorta or from the abdominal aorta (with the vessel penetrating the diaphragm to reach the sequestered lung). Surgical resection is usually indicated in virtually all cases of sequestration to treat persistent symptoms, prevent future complications, and/or to confirm the diagnosis [1, 4, 5].

Surgery for sequestration can be via either open thoracotomy or conventional multi-port Video-Assisted Thoracoscopic Surgery (VATS) [1–8]. Herein, we reported the first case – to the best of our knowledge – in which lobectomy for an intralobar pulmonary sequestration was performed using a uniportal VATS approach.

## Case presentation

A 34 year old male non-smoker presented with recurrent episodes of febrile pneumonia since childhood, usually requiring admissions for treatment with antibiotics. However, he was not investigated further at that time. The episodes had become slightly less frequent during adolescence, but were now becoming increasing frequent and severe over the past few years, prompting the patient to seek medical attention. It was noted that the patient had normal lung function, and had no history of any other medical conditions.

**Fig. 1:**
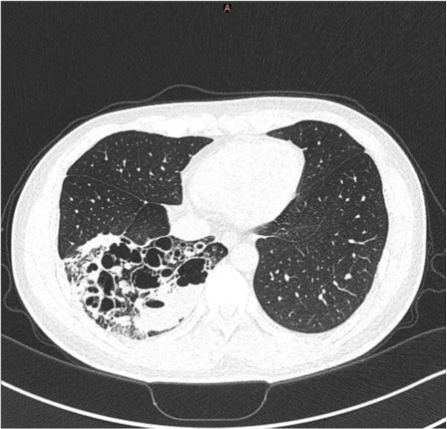
CT scan of the thorax (lung window) showed that the right lower lobe had been very extensively replaced by multi-cystic changes and diffuse inflammation. Other than sequestration, differential diagnoses at that time included CCAM and bronchiectasis

**Fig. 2:**
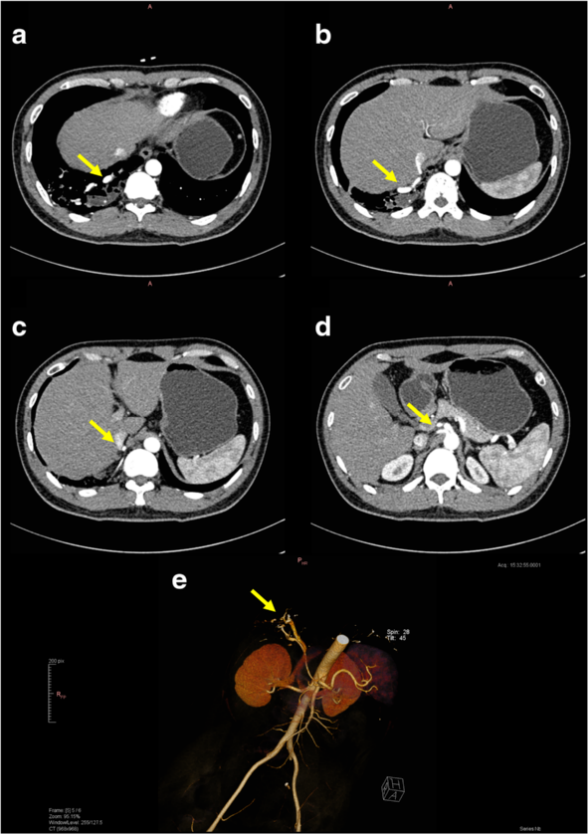
CT scanning with IV contrast confirmed the diagnosis of right lower lobe pulmonary sequestration by clearly demonstrating the aberrant feeding vessels (*yellow arrows*). **a**-**d** The entire course of the vessels from the abdominal aorta to the right lower lobe can be traced. **e** Detailed visualization from 3D reconstruction facilitates pre-operative planning

A CT scan of the thorax arranged by another unit showed that most of the right lower lobe of lung was destroyed and replaced by cystic changes with multiple fluid levels within (Fig. 1). Much of the remaining right lower lobe was also consolidated and inflamed, but all other lung lobes appeared normal. The initial working diagnosis was of congenital cystic adenomatoid malformation (CCAM). On attendance of our unit, a CT of the abdomen with IV contrast was performed to exclude any other associated congenital defect, and it was then discovered that the right lower lobe of lung received anomalous arterial supply from the abdominal aorta (Fig. 2). The diagnosis was revised to one of intralobar pulmonary sequestration. In view of the recurrent pneumonic episodes, surgery was offered.

**Fig. 3:**
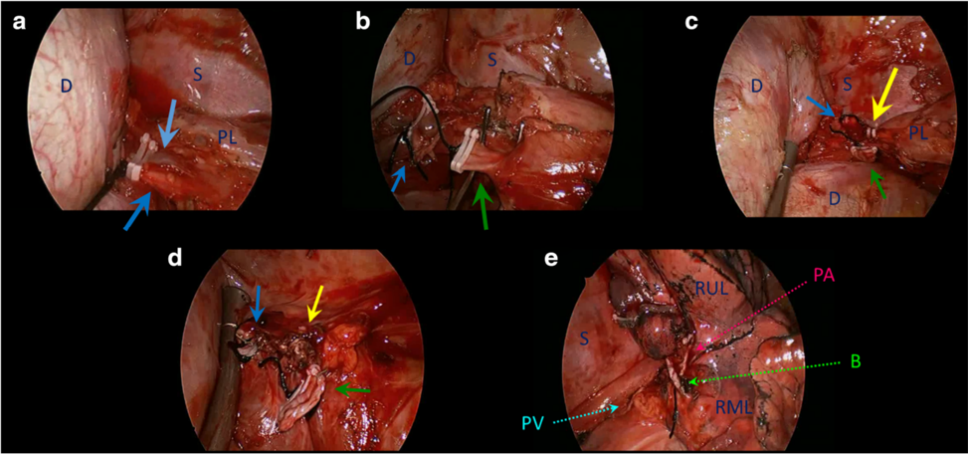
Intra-operative views (legend: D = diaphragm; S = spine; PL = pulmonary ligament; RUL = right upper lobe; RML = right middle lobe). **a** The first aberrant feeding vessel entered the chest by penetration through the posterior diaphragm. It promptly divides into two branches once in the chest (light and dark blue arrows respectively). This vessel has been secured proximally with silk ligature and double vascular clips prior to staple-division. **b** The second aberrant feeding vessel (green arrow) also entered the chest by penetration through the posterior diaphragm (blue arrow = stump of first aberrant vessel). This second vessel has been secured proximally with silk ligature and double vascular clips prior to staple-division. This view gives some impression of the extensive adhesiolysis that was required around the right lung base, diaphragm and spinal area in order to expose these aberrant feeding vessels. **c** The third aberrant feeding vessel (yellow arrow) entered the chest along the pulmonary ligament (blue arrow = stump of first aberrant vessel; green arrow = stump of second aberrant vessel). This small third vessel has been secured proximally with double vascular clips prior to division using an ultrasonic energy device. Notice that the diaphragm has been retracted using the suction to expose the vessels. **d** View of the stumps of all 3 aberrant feeding vessels following division (blue arrow = first vessel; green arrow = second vessel; yellow arrow = third vessel). **e** View of the hilum following Uniportal right lower lobectomy, showing the stapled stumps of the inferior pulmonary vein (PV), right lower lobar pulmonary artery (PA), and right lower lobar bronchus (B)

**Fig. 4:**
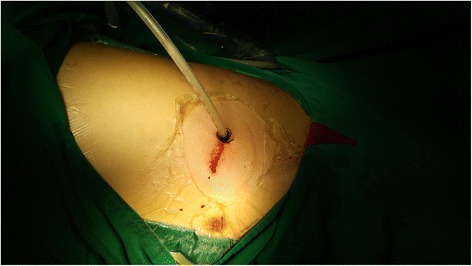
A 22F chest tube was placed via the Uniport at the end of the operation

A right lower lobectomy was performed using a uniportal VATS approach. Under general anesthesia and with pre-emptive paravertebral blockade, a 3.5 cm incision was made in the right 5th intercostal space between the anterior and mid-axillary lines. No rib-spreading was used, and an Alexis wound protector was applied (Applied Medical, Rancho Santa Margarita, CA). The entire operation was performed using a 30° 5 mm video-thoracoscope and conventional instruments (not dedicated uniportal instruments). Intra-operatively, extensive post-inflammatory adhesions were found over the entire postero-basal aspects of the right lower lobe. Careful release of these adhesions using a combination of energy devices (ultrasonic and diathermy) and long Metzenbaum scissors revealed three abnormal feeding vessels. Two larger feeding vessels entered from the abdomen into the thorax by penetrating the posteromedial diaphragm, with each approximately 8-10 mm in diameter (Fig. 3). One vessel branched soon after entering the thorax to enter the sequestration as a further two branches. Each of these larger abnormal feeding vessels was first proximally ligated with a strong silk ligature, then proximally clipped with two Hemo-o-Lock vascular clips (Weck Surgical Instruments, Teleflex Medical, Durham, NC), and then finally staple-divided using an Endo-GIA stapler with a Tristaple Curved Tip vascular reload (Covidien, Norwalk, CT). The third abnormal feeding vessel was a small (2 mm) arterial vessel found in the lower pulmonary ligament. This was proximally clipped with two Hemo-o-Lock vascular clips, and then divided using an ultrasonic energy device. Once these three abnormal feeding vessels were divided, the right lower lobectomy was completed using the standard uniportal technique [8], dividing the lobar vein, artery and bronchus in that order. The operation was completed in 228 min, and the blood loss was 60 ml. One 22 F chest tube was inserted via the uniport (Fig. 4), and this was connected to a digital chest drainage device (Medela AG, Baar, Switzerland) set to a negative pressure of -8 cm.H2O.

Post-operatively, the patient recovered uneventfully and was virtually pain-free whilst taking oral paracetamol (1 g 6-hourly) only. He was mobilize freely on the morning after surgery. There was no post-operative air leak, but the chest drain was removed on the third post-operative day because of initially high daily drainage of serous fluid. The patient was discharged home in good condition on the fourth post-operative day.

Histology of the resected lobe confirmed the diagnosis of an intralobar pulmonary sequestration.

## Conclusion

Despite being a benign condition, the potential complications of pulmonary sequestration are serious and may include recurrent sepsis, hemoptysis, and congestive heart failure [1–4]. Resection of the sequestrated lung is, therefore, the definitive treatment of choice once a diagnosis is made. Furthermore, sequestration can often be difficult to diagnose with pre-operative diagnosis achieved in only 12–58 % [2, 3]. Hence, surgery is also often performed with an exploratory intent in patients suspected to have sequestration.

Pulmonary sequestration is classified into extralobar or intralobar types [1, 3, 6]. In the extralobar variant, the sequestrated lung tissue is completely separated from the normal lung and has its own visceral pleura covering. Because extralobar sequestration essentially constitutes a discrete mass apart from the lung, resection is relatively straightforward, with comparatively easy identification and division of the abnormal feeding vessels followed by resection of the mass without need to remove any normal lung tissue [9]. The intralobar variant is more common, accounting for 75–84 % of all pulmonary sequestrations [3, 6]. With intralobar sequestration, the abnormal lung is surrounded by normal lung and has no separate pleura investment. The intralobar variant is regarded as technically more challenging to resect given that: the abnormal feeding vessels are more difficult to locate; recurrent infection causing pleural adhesions is more common; and it is necessary to remove the surrounding normal lung which is inseparable from the sequestration. Surgery for intralobar sequestration usually requires a lobectomy or segmentectomy, after the abnormal feeding vessels are divided. Such surgery for pulmonary sequestration can be performed via open thoracotomy or using a VATS approach [1, 3]. For the latter, a variety of techniques have been reported, including the conventional 3-port technique, as well as variants using 2- or 4-port techniques [2, 6, 7, 10].

In our patient, the operation could be considered relatively challenging from a technical standpoint. There were not one, but three abnormal vessels from the abdominal aorta feeding the sequestrated lung. These were of various sizes and entered the thorax via different routes. The patient was also an adult and had a long history of recurrent episodes of infection. This meant the presence of extensive post-inflammatory adhesions, further making the identification of the abnormal feeding vessels difficult. Failure to identify and safely divide these vessels could result in catastrophic hemorrhage [1]. Furthermore, the size of the sequestrated lung was relatively large, necessitating a complete lobectomy. Nonetheless, despite these hurdles, a Uniportal thoracoscopic approach was successfully used to complete the lobectomy in this patient. The relatively longer operation time compared to our previously reported experience is indicative of the technical difficulty in this case rather than with the Uniportal approach itself. To date, only one previous report exists, to our knowledge, of a Uniportal approach for the treatment of pulmonary sequestration [9]. However, that was a case of a small extralobar sequestration in a patient with no noted history of prior infections or symptoms. We believe that our case showcases the safety and feasibility of the Uniportal approach for sequestration surgery even in technically challenging situations. No expensive surgical instruments specifically designed for Uniportal surgery were required.

Our experience with this case has taught us that when considering the use of the Uniportal approach for a patient, it is especially important to plan surgery carefully. The differential diagnosis of an intralobar sequestration may include a number of congenital cystic lung conditions (especially CCAM which can mimic sequestration clinically as well as radiologically), as well as acquired disease such as focal bronchiectasis (which the initial CT in our patient also suggested as a possible differential diagnosis). In our patient, the decision to perform the CT of the abdomen with IV contrast proved critical. CT allowed accurate diagnosis of a sequestration by visualization of the aberrant feeding vessels [11]. This in turn allowed a suitably cautious dissection of the extensive post-inflammatory adhesions, and avoiding of inadvertent – and disastrous – damage to the abnormal feeding vessels. Given the size of the two larger feeding vessels in this patient, we also chose to err of the side of over-caution in securing them before division: using a combination of ligation and double vascular clipping proximally prior to application of the endostapler.

In recent years, a good number of authors have suggested potential advantages of using the Uniportal approach. These claims have included: reduced pain and faster recover after surgery; restriction of neuralgia and paresthesia to only one intercostal space; and better visualization and operative geometry [8, 12, 13]. Although we have performed Uniportal lobectomy routinely for lung cancer for a few years now, we make no such claims for the benefit of this approach for sequestration surgery as our experience is limited to just this one case thus far. We only conclude at this time that the approach can be safely used, and remains an option for the experienced surgeon.

## Consent

Written informed consent was obtained from the patient for publication of this report and any accompanying images.
